# Heterochronic Expression of TFAP2β in Coarctation of the Aorta

**DOI:** 10.21203/rs.3.rs-10021035/v1

**Published:** 2026-06-30

**Authors:** Rick Mathews, Rebecca Ditmore, Victoria Krajbich, Monica T Hinds, Randall L Woltjer, Ashok Muralidaran

**Affiliations:** Oregon Health & Science University; Oregon Health & Science University; Oregon Health & Science University; Oregon Health & Science University; Oregon Health & Science University; Oregon Health & Science University

**Keywords:** coarctation, aorta, TFAP2β, ductus arteriosus

## Abstract

Coarctation of the aorta (CoA) is an anatomically defined lesion with a surgical solution and no established mechanistic etiology. TFAP2β has demonstrated specificity to ductal smooth muscle cells in CoA. In mammalian models, ductal TFAP2β protein expression declines across the third trimester, with protein undetectable at or near term in mice and rats as well as corroborating mRNA decline in non-human primates. We report robust postnatal TFAP2β protein colocalized to the elastin-poor obstructive shelf in a surgical CoA resection.

## Case Report

An early term (37 weeks 0 days) dichorionic diamniotic neonate with severe intrauterine growth restriction (birthweight 1.4 kg; weight at presentation 2.1 kg) was diagnosed with coarctation of the aorta (CoA) and patent ductus arteriosus (PDA) on the sixth day of life. Transthoracic echocardiography demonstrated discrete juxtaductal narrowing with a small patent foramen ovale and no other significant intracardiac anomalies. Trio rapid whole-genome sequencing was non-diagnostic. Prostaglandin infusion was initiated to maintain ductal patency and at 29 days of life, the infant underwent extended end-to-end anastomotic repair for CoA. The resected CoA segment was fixed in 10% formalin, paraffin embedded and serially sectioned at 5 μm under IRBs STUDY00022719 and STUDY00027302.

**Figure 1:**
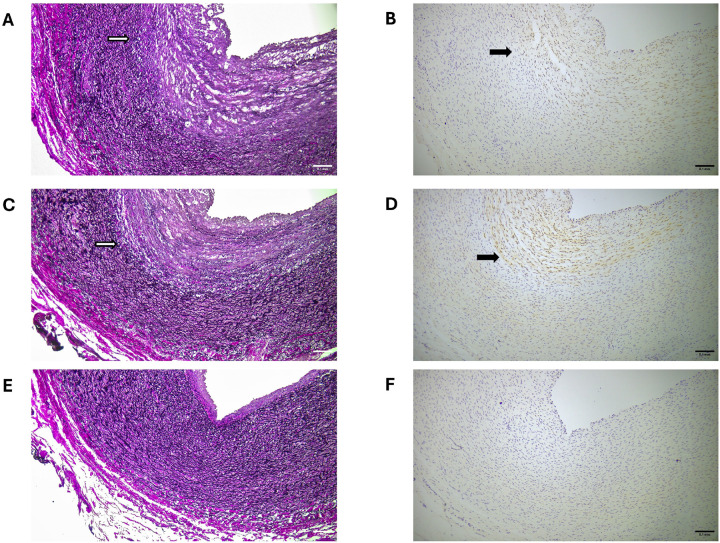
Elastin Histology and TFAP2β Immunohistochemistry in a Surgical Resection of Coarctation of the Aorta Verhoeff van Gieson staining of a resected neonatal coarctation specimen at multiple levels (**A, C, E**) demonstrates well arranged, concentric elastic lamellae of the aorta relative to the disarray of fragmented elastin fibers and intimal cushion of the coarctation obstruction. The boundary between elastin rich and poor regions of the CoA resection is marked (arrows) and there is specific and robust staining of the transcription factor AP2β (**B, D, F**) colocalizing with the elastin poor staining. VVG images were taken under default settings, saturation, contrast, and brightness were optimized for representative images matching eye piece visualization of AP2β immunohistochemistry.

Verhoeff van Gieson staining (Figure 1 A, C, and E) demonstrated an abrupt transition from the organized, concentric elastin lamellae of the aorta to the disorganized and elastin poor CoA obstructive shelf. To assess the molecular identity of the elastin poor tissue, immunohistochemistry (IHC) for TFAP2β (1:150, HPA034683, Atlas Antibodies/ Sigma Aldrich), known to selectively stain the ductus arteriosus in CoA resections[1], demonstrated robust positive staining colocalized to the elastin poor obstructive shelf of the CoA resection (Figure 1 B, D, and F). Adjacent aortic territory remained negative for TFAP2β expression. These data corroborate ductal selective expression of TFAP2β in CoA patients in multiple, independent clinical cohorts[1,2].

## Discussion

A crucial interpretive gap exists in this literature. TFAP2β in the ductus arteriosus demonstrates declining expression across the third trimester[3–5]. This is consistent across multiple rodent studies in which protein expression is not detected at or near term[3,5], with corroborating mRNA data in baboons[6]. Parikh et al demonstrate relatively higher mRNA expression of TFAP2β in the ductal vs the aortic portion of CoA resections from patients between six days to one year of age[2]. Iwaki et al demonstrate selective TFAP2β IHC in the ductal smooth muscle cells and intimal shelf of CoA resections in a study of nine CoA patients between eight days to nine months of age[1]. These windows extend beyond any developmental timeframe in which TFAP2β protein is detectable in cross-species mammalian data[3,5] and beyond any plausible duration of perinatal prostaglandin exposure. Furthermore, Parikh et al consider patients with and without PDA or prostaglandin exposure[2] and Iwaki et al explicitly exclude patients exposed to prostaglandin yet observing selective TFAP2β protein expression in CoA resections.

Postnatal detection of TFAP2β protein in CoA resections may not merely be a marker of ductal-origin tissue but evidence of heterochronic protein expression well beyond currently evident mammalian developmental windows[3–6]. At the tissue architecture level, the heterochronicity may be attributable to compartment boundary failure[7], persistence of fetal architecture[8], or other unknown mechanisms. Cellularly, the heterochronicity may be attributable to an arrested fetal transcriptional state, maladaptation, dedifferentiation[9], or other unknown mechanisms. Gestational architectural studies of the ductal aortic junction[8] are warranted for developmental context. Spatial transcriptomic and proteomic analyses are warranted for further mechanistic differentiation.

Limitations of this study include a single clinical sample; however, this observation adds to evidence from multiple, independent clinical cohorts[1,2]. This study does not confirm downstream activation of TFAP2β targets, which may differ between fetal and postnatal as well as physiologic and pathologic contexts.

Taken together, mammalian data demonstrate ductal TFAP2β mRNA expression peaks during the second trimester and decreases across the third trimester[3,4,6] with no protein expression detected at or beyond term in mammalian models examined to date[3,5], explicitly questioning why protein expression is selectively and heterochronically detectable in the elastin poor obstructive shelf of postnatal CoA patients[1] in multiple, independent clinical cohorts and further questioning whether persistent expression of TFAP2β in rodent models would replicate CoA pathology.

## Data Availability

All raw data available upon reasonable request to the corresponding author.
